# Placental Protein 13 and Syncytiotrophoblast Basement Membrane Ultrastructures in Preeclampsia

**DOI:** 10.3390/medicina60071077

**Published:** 2024-06-30

**Authors:** Peby Maulina Lestari, Noroyono Wibowo, Damar Prasmusinto, Muhammad Yamin, Nuryati Chairani Siregar, Joedo Prihartono, Ina Susianti Timan, Johanes C. Mose, Iche Andriyani Liberty, Cindy Kesty, Bella Stevanny

**Affiliations:** 1 Department of Obstetrics and Gynecology, Dr. Mohammad Hoesin General Hospital, Faculty of Medicine, Universitas Sriwijaya, Palembang 30114, Indonesia; cindykestyjl18@gmail.com; 2 Department of Obstetrics and Gynecology, Dr. Cipto Mangunkusumo National General Hospital, Faculty of Medicine, Universitas Indonesia, Jakarta 10430, Indonesia; norowibowo@gmail.com (N.W.); masdamar@yahoo.com (D.P.); 3 Department of Cardiology, Dr. Cipto Mangunkusumo National General Hospital, Faculty of Medicine, Universitas Indonesia, Jakarta 10430, Indonesia; muhyam511@gmail.com; 4 Department of Anatomical Pathology, Dr. Cipto Mangunkusumo National General Hospital, Faculty of Medicine, Universitas Indonesia, Jakarta 10430, Indonesia; anisiregar@gmail.com; 5 Department of Community Medicine, Dr. Cipto Mangunkusumo National General Hospital, Faculty of Medicine, Universitas Indonesia, Jakarta 10430, Indonesia; joedopri@gmail.com; 6 Departement of Clinical Pathology, Dr. Cipto Mangunkusumo National General Hospital, Faculty of Medicine, Universitas Indonesia, Jakarta 10430, Indonesia; ina_st_ui@yahoo.com; 7 Department of Obstetrics and Gynecology, Dr. Cipto Mangunkusumo National General Hospital, Faculty of Medicine, Universitas Padjadjaran, Bandung 45363, Indonesia; jcmose07@yahoo.com; 8 Department of Public Health and Community Medicine, Faculty of Medicine, Universitas Sriwijaya, Palembang 30114, Indonesia; iche.aliberty@gmail.com; 9 Tropical Diseases Research/World Health Organization Fellow, Infectious Diseases Data Observatory, University of Oxford, Oxford, ON OX1 4BH, Canada; 10 National Task Force of Reproductive Tract Infection, Indonesian Society of Obstetrics and Gynecology, Jakarta 10320, Indonesia

**Keywords:** endothelial dysfunction, Placental Protein 13, preeclampsia, syncytiotrophoblast basement membrane

## Abstract

*Background and Objectives*: Preeclampsia has been linked to an inflammatory response that may be brought on by endothelial cell dysfunction. This paper investigates the pathomechanism of syncytiotrophoblast basement membrane (STBM) damage and Placental Protein 13 (PP13) release, which may have a role in systemic endothelial dysfunction in preeclampsia. *Materials and Methods*: This comparative cross-sectional study involves 54 preeclampsia patients (27 early-onset preeclampsia and 27 late-onset preeclampsia) and 27 pregnant women with normal blood pressure. An enzyme-linked immunosorbent assay was performed to evaluate maternal blood levels of PP13. Following birth, a portion of the placenta was collected for transmission electron microscope (TEM) and immunohistochemical (IHC) analysis. The data were analyzed using STATA version 15. *Results*: PP13 expression in the placental syncytiotrophoblast was significantly lower in the early-onset preeclampsia, compared to late-onset preeclampsia and normotensive pregnancy, group (*p* < 0.001). In contrast, serum PP13 levels were found to be the highest in the early-onset preeclampsia group, although no significant difference were found in mean maternal serum levels of PP13 between the three groups. The decreased PP13 expression in placental syncytiotrophoblast can be attributed to the greater extent of damage in the STBM in early-onset preeclampsia that leads to the release of a larger amount of PP13 into maternal circulation. The hypothesis aligns with the TEM analysis results. Preeclamptic pregnancies showed placental syncytiotrophoblast aponeurosis, whereas normotensive pregnancies did not. Placental lesions and STBM shedding were found to be more pronounced in early-onset preeclampsia compared to late-onset preeclampsia. *Conclusions*: PP13 and STBM damage may play a role in systemic endothelial dysfunction in preeclampsia.

## 1. Introduction

Preeclampsia is generally defined as the onset of hypertension and proteinuria in previously normotensive women after 20 weeks of pregnancy [1,2]. More than 50,000 maternal fatalities and over 500,000 fetal deaths occur yearly due to this condition, accounting for 2 to 8% of pregnancy-related problems [3]. Preeclampsia can classified into early-onset (20–34 weeks) and late-onset (>34 weeks) preeclampsia [2]. The main pathogenesis of preeclampsia is failure of remodeling of the spiral arteries in the placenta which causes endothelial dysfunction, which occurs after complete transformation of the subplacental arteries at 20–24 weeks of gestation [4].

Endothelial cell dysfunction in preeclampsia can result from the activation of factors originating from the fetus, mother, and also placenta. An elevation in trophoblast aponecrosis material leads to a rise in fetal DNA levels and the presence of proteins such as alpha activin, transferrin, placental alkaline phosphatase, activin A, inhibin A, and cytokeratin. These factors, together with additional factors secreted from the placenta (referred to as factor X by Redman and Sargent), are released to the circulation and trigger endothelial cell dysfunction [5]. Placental Protein 13 (PP13) is a placenta-specific protein produced solely by the syncytiotrophoblast in the placenta and not by any other tissues. Decreased PP13 expression in the placental syncytiotrophoblast and low serum concentration of PP13 in the first trimester are said to be predictive for the incidence of preeclampsia in the second half of pregnancy. It is estimated that placental ischemia that occurs in preterm preeclampsia will cause shedding of PP13 microparticles into the maternal circulation [6].

Apoptosis, a well-established cellular death mechanism, is a natural process of trophoblast turnover that commonly takes place in the placenta. The apoptotic materials consisting of accumulation of apoptotic syncytiotrophoblast cell nuclei are then wrapped in a vesicle membrane and form syncytial knots protruding from the apical membrane. These knots will then be released into maternal circulation. This release of apoptotic material normally does not elicit maternal inflammatory reaction. In preeclampsia, there is a change in the balance between proliferation, fusion, and apoptosis of trophoblast villi, leading to necrosis in several nonapoptotic areas of syncytiotrophoblast. The term used to describe this phenomenon is aponecrosis [7,8]. Differentiation of trophoblast villi in early pregnancy in preeclampsia will cause aponecrosis to replace the normally occurred apoptosis process [9], which cause increased release of the free trophoblast fragment called syncytiotrophoblast basement membrane (STBM) [10]. The STBM, once released, can pass through the lungs and can easily be detected in peripheral blood. Most importantly, it has the ability to induce systemic alterations in the maternal endothelium and immune system. Along with the release of STBM and necrotic cells into the maternal circulation, PP13 is also released [11]. It is hypothesized that an increase in aponecrosis and STBM release into the maternal circulation will accelerate the activation of maternal endothelial cells, and ultimately will cause endothelial dysfunction [12].

Huppertz and Kingdom evaluated the amounts of PP13 in the STBM of preeclamptic patients in their placental immunohistochemistry study and found that STBM is likely a means of transporting significant amounts of PP13 (formerly confined in the disrupted trophoblast), which might cause PP13 to rise in preeclampsia patients as gestational age increases [13]. Another study contends that the shedding of PP13-containing syncytiotrophoblasts in response to low levels of PP13 in the first trimester causes a rise in PP13 in the third trimester in preeclampsia patients [14].

The pathomechanism of STBM injury and PP13 release, which can lead to systemic endothelial dysfunction, is being examined in this study. This study aims to compare the level of serum PP13, PP13 expression in placental syncytiotrophoblast, and the ultrastructure of the placental STBM in the third trimester of normotensive pregnancy, early-onset preeclampsia, and late-onset preeclampsia.

## 2. Materials and Methods

### 2.1. Study Population

This is a comparative cross-sectional study using a consecutive sampling strategy involving 54 preeclampsia patients (27 early-onset preeclampsia and 27 late-onset preeclampsia) and 27 pregnant women with normal blood pressure. The minimum sample size was calculated using the sample size estimation formula for analytical studies with numerical data to determine the mean difference between two independent groups. The minimum sample size was calculated using confidence level of 95% (α = 0.05, zα = 1.96), power level of 90% (β = 0.1, zβ = 0.84), variability of 1.2, and effect size of 133.1 from previous study by Zadeh et al. [15] Recruitment of study subjects was carried out using the consecutive sampling method. The population of the study were all pregnant women who visited Dr. Mohammad Hoesin Hospital Palembang and Hermina Hospital Palembang with severe preeclampsia between October 2017 and January 2018. The inclusion criteria included pregnant women with intrauterine singleton pregnancy and severe preeclampsia—early onset (28–34 weeks) or late onset (>34 weeks). Given the uncertainty regarding the precise timing of preeclampsia development, delivery before 34 weeks of gestation was considered as an indicator of early-onset preeclampsia [16]. The control group consisted of expectant mothers with gestational ages over 37 weeks who were normotensive and were not yet in labor. The exclusion criteria included pregnant women with multiple gestation and those who refused to participate in the study. Subjects with history of heart or cardiovascular disease, prior kidney disease, impaired liver function, hematological disease, coagulation disorders, DIC, anticoagulant use, chronic antihypertensives use, or thrombolytics use were also excluded from the study.

### 2.2. Data Collection

Basic subject characteristics were gathered from medical records and histories. The blood sample and placental tissue were collected at the third trimester of pregnancy, with gestational age of 28–34 weeks for early-onset preeclampsia, >34 weeks for late-onset preeclampsia, and >37 weeks for normotensive pregnant women. The Human PP13 ELISA kit (BioVendor Research and Diagnostic^®^, Asheville, NC, USA) was used to quantify serum PP13 level. The blood sample was divided into serum separator tubes (SSTs) containing 6 milliliters of blood and centrifuged for around 10 min at 1500 rpm. A few parts of the placenta were taken after birth. Five different regions of the placenta of 1 × 1 cm in size were sampled at their entire thickness. Each site provided one piece of the sample, which was washed with physiological buffer to remove serum proteins that might clot and possibly hide surface layer details. It was then cut at approximately 4 μm and left at 4 °C for 4–12 h as a paraffin block at the Department of Anatomical Pathology of Dr. Mohammad Hoesin Hospital Palembang before it was stained with anti-PP13 antibody (Anti Galectin 13 antibody/ab218411, ABCAM^®^, Cambridge, MA, USA), for further analysis of the expression of PP13 in placental syncytiotrophoblast. Using a Nikon Eclipse 80i microscope for the immunohistochemical analysis, ImageJ was used for the computation. The data were interpreted by two anatomical pathologists. Each area was given 3 points for strong (deep brown), 2 points for moderate (light brown), 1 point for weak (bluish purple), and 0 points for no staining (purple) staining intensity. Immunostaining density value ranged from 4 points for 81–100% to 3 points for 51–80%, 2 points for 11–50%, 1 points for 1–10%, and 0 points for 0%, of positively stained cells. Immunohistochemical score was then calculated by multiplying immunostaining intensity value with immunostaining density value. Each sample underwent five visual field examinations before its color absorption compared to the anti-PP13 antibodies evaluated. Five full-thickness parts of the placenta, side by side with the part previously taken, were placed in a tube with a physiological buffer solution that had been prepared previously, stored at a temperature of 10 °C, and then examined using a transmission electron microscope (JEOL TEM-1010, Peasbody, MA, USA) at Eijkman Institute, Jakarta, to examine the syncytiotrophoblast aponecrosis in the placenta.

Written informed consent was obtained from each participant woman before their inclusion in the study. Ethical permission was obtained for the study from the Ethics Committee of the Faculty of Medicine, University of Indonesia (953/UN2.F1/ETIK/2017).

### 2.3. Analytical Statistics

STATA version 15 (College Station, TX, USA) was used to analyze the data. Independent *T*-test and Mann–Whitney were used to compare quantitative data between groups. For statistical analyses involving more than two groups, one-way ANOVA and Kruskal–Wallis were used. The significance level for the data was established at *p* < 0.05, with a 95% confidence interval.

## 3. Results

### 3.1. Characteristics of Research Subjects

A total of 81 subjects was included in the study. Twenty-seven people made up the control group (normotensive pregnancy), 27 people made up the early-onset preeclampsia group, and 27 people made up the late-onset preeclampsia group in this sample. In the third trimester of pregnancy, differences in maternal age, height, body surface area (BSA), body mass index (BMI), and parity between early-onset, late-onset preeclampsia, and normotensive pregnancies were not statistically significant (Table 1).

### 3.2. Concentration of PP13 in Maternal Serum

In this study, maternal blood levels of PP13 were assessed in comparison to controls in both early-onset and late-onset preeclampsia. The results are shown in Table 2. According to the research, third-trimester pregnancies with early-onset preeclampsia, late-onset preeclampsia, and normotensive pregnancies were not statistically different in their PP13 levels. However, the mean PP13 levels were comparatively higher in early-onset preeclampsia compared to late-onset preeclampsia and controls. 

### 3.3. Expression of PP13 in Placental Syncytiotrophoblast

The expression of PP13 in the placenta was evaluated using the IHC analysis. Anti-PP13 antibodies were used for staining. In the third trimester of pregnancy, the expression of PP13 in placental syncytiotrophoblast differed significantly between early-onset preeclampsia, late-onset preeclampsia, and normotensive groups (Table 3).

### 3.4. Placental Aponecrosis

**Figure 1:**
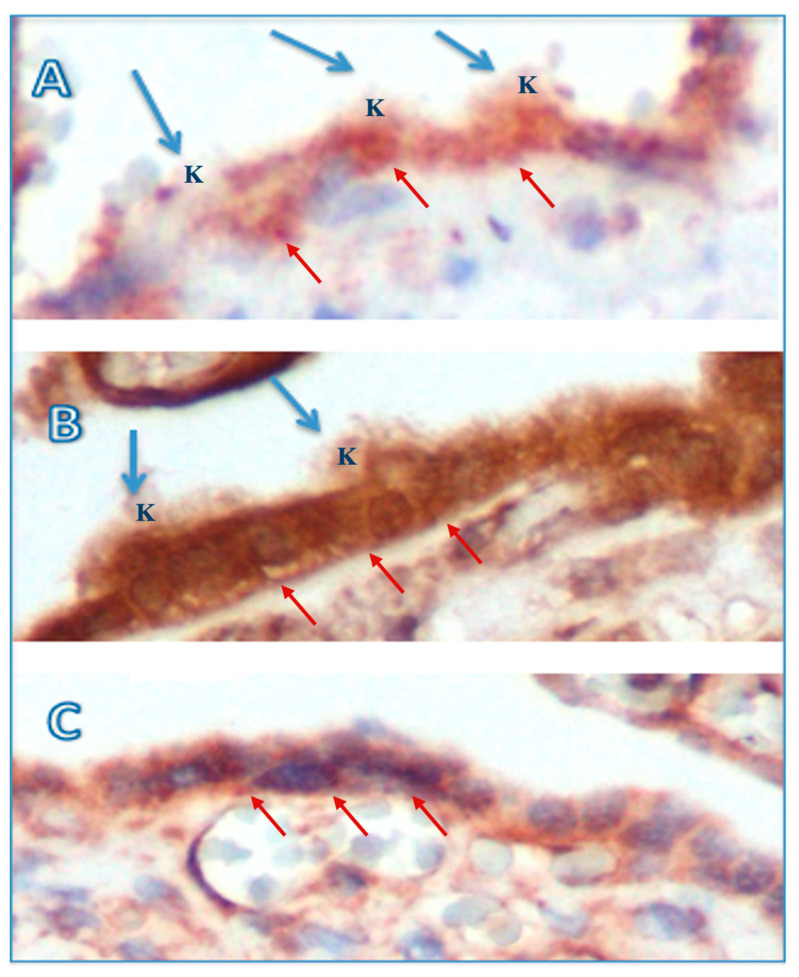
Syncytiotrophoblast basement membrane on PP13 immunohistochemistry examination (original magnification 400×). Anti-PP13 antibody was used for the immunostaining of the apex of the syncytiotrophoblast basement membrane. (**A**) Early-onset preeclampsia: irregular basement membrane (red arrow) with multiple syncytial knots (blue arrow) on the brush border membrane prepared to be released into the maternal circulation. (**B**) Late-onset preeclampsia: basement membrane (red arrow) with fewer syncytial knots (blue arrow) compared to in early-onset preeclampsia. (**C**) Control: an intact and normal basement membrane (red arrow). K: syncytial knot.

As shown in Figure 1, preeclampsia caused alterations in the brush border of the syncytiotrophoblast membrane.

**Figure 2:**
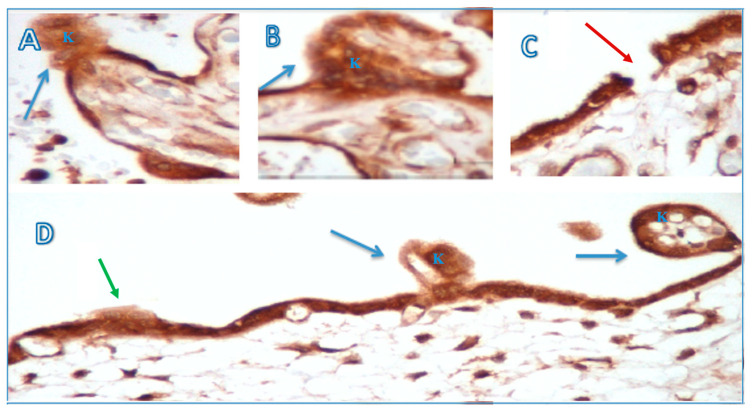
Syncytial knots on the surface of the syncytiotrophoblast basal membrane with anti-PP13 antibody staining (original magnification 400×). (**A**,**B**) Syncytial knots (blue arrow) ready to be discharged into the maternal circulation. (**C**) A breached syncytiotrophoblast apical membrane (red arrow), suggesting that the syncytial knot has entered the maternal circulation. (**D**) Three phases of damage basement membrane syncytiotrophoblast damage, from minimal damage (green arrow) to the forming of syncytial knots (blue arrow). K: syncytial knot.

Figure 2 shows that the preeclampsia group had protrusions at the apex of the basement membrane brought on by necrosis or syncytial knots, and that a normal syncytiotrophoblastic basement membrane was seen in IHC investigation.

**Figure 3:**
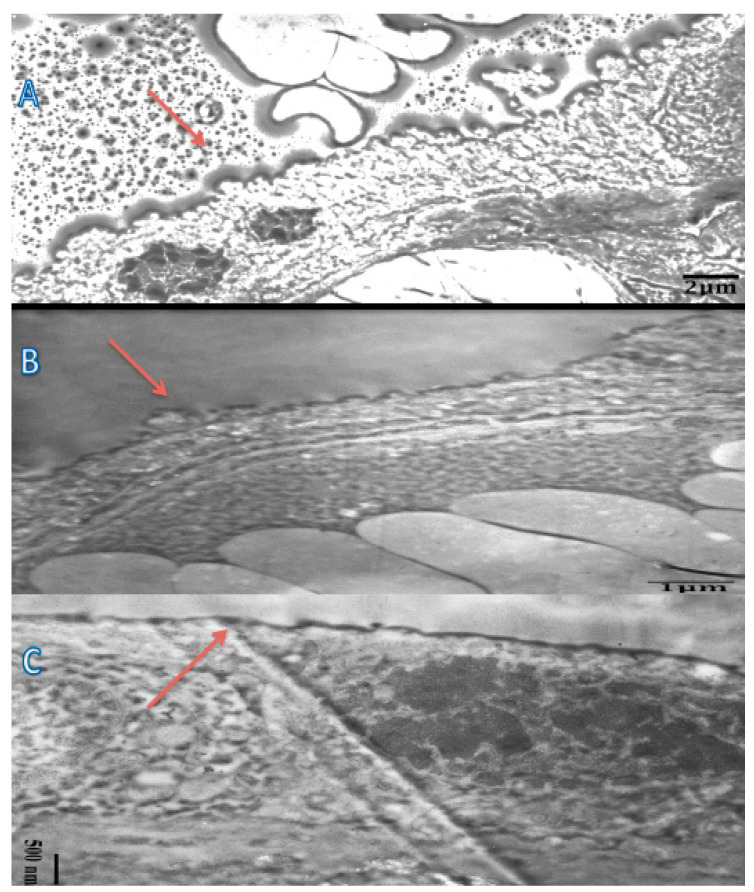
Transmission electron microscope (TEM) images of brush border membrane (red arrow) of the syncytiotrophoblast (original magnification 2500×, 80 kv). (**A**) Early-onset preeclampsia: The brush boundary membrane is wavy and irregular with a discontinuous look in certain areas. (**B**) Late-onset preeclampsia: The brush border membrane is likewise uneven with irregular smaller waves. (**C**) Normotensive pregnancy: The brush boundary membrane has a flat, uniform surface. JEOL 1010 transmission electron microscope, mag. 2500×, 80 kv.

To support the notion of damage and aponeurosis in the syncytiotrophoblastic basement membrane, a TEM investigation was carried out in addition to the immunohistochemical test. Figure 3 depicts the basal membrane as a whole under TEM inspection. Preeclampsia showed a more pronounced variation in the appearance of the basement membrane compared to normotensive pregnancy groups.

**Figure 4:**
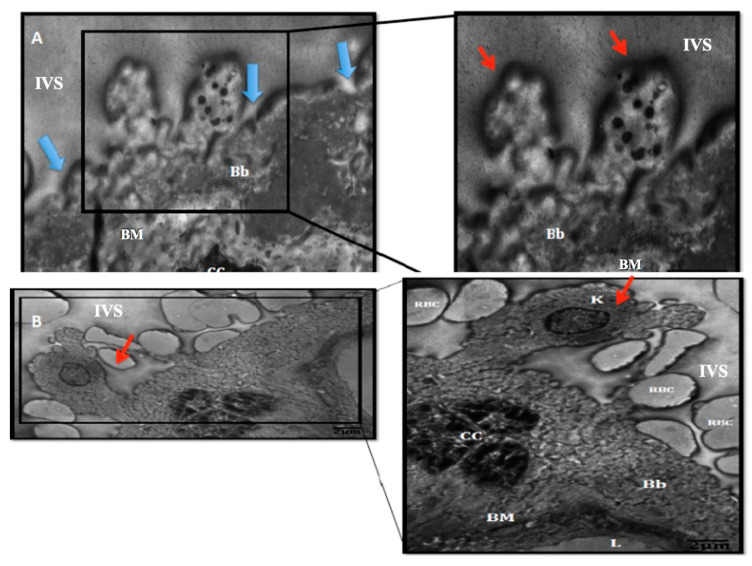
Syncytial knots in early-onset preeclampsia and late-onset preeclampsia (original magnification 2500×, scale bar 2 μm). Syncytial knots (red arrows) are easier to find in early-onset preeclampsia (**A**) compared to in late-onset preeclampsia (**B**). Also note the brush border membrane, which is interrupted in early-onset preeclampsia (blue arrows). Bb: brush border, CC: chromatin condensation, BM: basal membrane, K: syncytial knot, L: fetal capillary lumen, IVS: intervillous space or maternal blood space, RBC: maternal red blood cell.

A comparison of syncytial knots in early-onset preeclampsia and late-onset preeclampsia is depicted in Figure 4.

**Figure 5:**
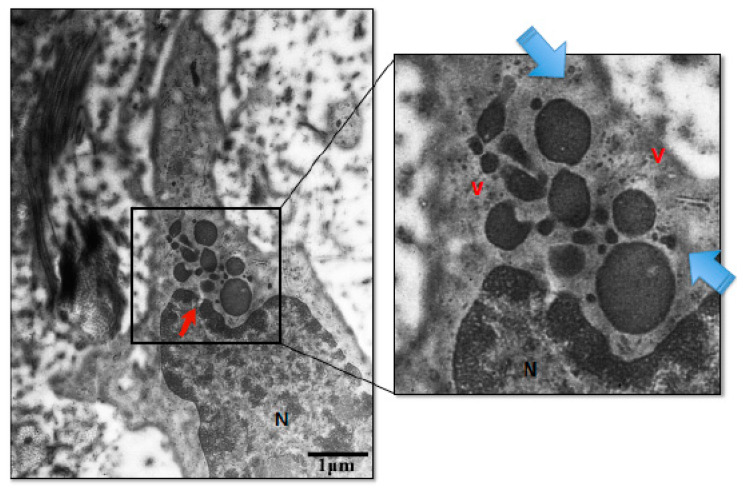
Wave like-aponecrosis shedding (original magnification 2500×, scale bar 1 μm). An electron microscope examination showed condensation of nuclear chromatin that accumulated (red arrow) to form knots protruding from the brush border. At the same time, the surrounding cytoplasm was edematous (blue arrow) and accompanied by fibrinous and other necrotic materials (v). This material is ready to be released into the maternal circulation. N: nucleus.

Glimpses into the process of wave-like aponecrotic shedding are shown in Figure 5.

## 4. Discussion

### 4.1. Characteristics of Research Subjects

This study found that body weight and BMI, two factors that impact preeclampsia incidence, did not appear to differ substantially between early-onset and late-onset preeclampsia, suggesting that obesity may not be a risk factor for early-onset preeclampsia. This investigation’s findings align with Iacobelli et al.’s [17] study findings, which found that although a significant difference in BMI was found between preeclampsia and healthy controls, there was no distinction between early-onset and late-onset preeclampsia. However, this difference was not statistically significant.

### 4.2. Maternal Serum Concentration of PP13

In this study, the third-trimester serum concentration of PP13 in early-onset preeclampsia was higher (260.8 (38.4–1233.4) ng/mL) compared to late-onset preeclampsia (244.2 (80.4–722.6) ng/mL) and the control group (232.5 (34.6–476) ng/mL); however, the difference was not statistically significant. These findings are consistent with cross-sectional research by Than et al. [14] showing that serum PP13 levels were higher in early-onset preeclampsia compared to late-onset preeclampsia in the third trimester of pregnancy, although no significant differences were found between late-onset preeclampsia and controls.

Previous research reported that the median maternal serum PP13 level in healthy pregnant women rises from 166 pg/mL in the first trimester to 202 pg/mL in the second and 382 pg/mL in the third trimester [18]. Compared to normotensive pregnancies, maternal serum PP13 levels in preeclampsia were lower in the first trimester (MoM 0.14) and considerably greater in the third trimester (MoM 1.79) of pregnancy. Other investigations have demonstrated that the maternal blood concentration of PP13 in partial HELLP syndrome is 213.0 (74–571) (*p* = 0.01) and in early-onset preeclampsia is 250.0 pg/mL (52–462) (*p* = 0.02), and statistically significant when compared to the control group (109.6 (31–203)) [19]. In pregnant women with preeclampsia, maternal PP13 serum concentrations in the first trimester of pregnancy tend to be lower. Maternal PP13 concentrations then to increase near delivery, proving that PP13 is a substance specifically linked to pregnancy and the placenta [18]. Given that there was no statistically significant change in maternal PP13 serum concentrations throughout the third trimester of pregnancy in this investigation, additional variables may contribute to PP13 in the latent phase of preeclampsia. A drop in PP13 concentrations observed during the first trimester of pregnancy has been linked to an increased risk of preeclampsia [20,21]. Low PP13 levels during the first trimester may last the whole pregnancy, preventing a significantly higher level of PP13 in preeclampsia compared to normal pregnancy despite the excessive release of PP13 particles into the bloodstream during the aponeurosis and STBM shedding. Previous research has also demonstrated a rise in villous trophoblastic necrosis cells in the maternal circulation during the third trimester of normal pregnancy [18,22]. Because of this, there could not be a discernible difference in PP13 concentrations between preeclampsia and normal pregnancy throughout the third trimester of pregnancy. 

In this study, the relatively lower serum PP13 level in the third trimester of the early-onset preeclampsia group might be due to the greater extent of damage in the STBM that leads to the release of a larger amount of PP13 into maternal circulation. The hypothesis aligns with the TEM analysis results.

### 4.3. The Expression of PP13 in Placental Syncytiotrophoblast

PP13 expression is downregulated in the placentas of individuals diagnosed with preeclampsia. Nevertheless, the specific processes accountable for this phenomenon have yet to be established. Given the direct connection between the syncytiotrophoblast and maternal blood, it is reasonable to consider that a reduced synthesis of PP13 could explain the lower levels of this protein in the first-trimester maternal serum of individuals who are likely to develop preeclampsia. The lower concentration of PP13 is detected during the first trimester of pregnancy, months before the clinical disease develops [23]. However, after complete transformation of the subplacental arteries at 20–24 weeks of gestation, there is failure of remodeling of the spiral arteries in the placenta [4]. This abnormality causes damage to occur in the syncytiotrophoblast basement membrane and increased aponecrosis, causing more PP13 to be released to the maternal circulation and even lower expression of PP13 in the placenta of preeclamptic patients. 

Through immunohistochemistry (IHC) examination using anti-PP13 antibodies, researchers tried to assess the expression of PP13 in the placental syncytiotrophoblast. The IHC score is a score used to assess the intensity of staining in the syncytiotrophoblast. The smaller the IHC score, the less the tissue absorbs the stain and the less expression of PP13 there is in the syncytiotrophoblast basement membrane. In this study, the IHC score in early-onset preeclampsia was significantly lower than the IHC score in late-onset preeclampsia and normotensive pregnancy (3.38 ± 0.46; 4.06 ± 0.82; 4.51 ± 1.16; *p* < 0.05). PP13 expression, which was lower than the other two groups, was most likely due to the damage that occurred in the syncytiotrophoblast basement membrane, which was more extensive than in late-onset preeclampsia and the control group so that a lot of PP13 was released into the maternal circulation. These results are in line with research conducted by Than et al. [14], and reinforce the previous theory that aponeurosis occurs in preeclampsia, especially in early-onset preeclampsia.

### 4.4. Placental Syncytiotrophoblast Basement Membrane Damage

Aponeurosis is the programmed cell death that is thought to occur in preeclampsia. It is estimated that the more aponeurosis that occurs in the placenta, the faster and more severe the degree of preeclampsia that occurs. With the occurrence of aponeurosis, necrotic tissues, and microparticles, including PP13, particles will be released and form syncytiotrophoblast syncytial knots. This material is then released into the maternal circulation, and this is what is expected to stimulate a maternal inflammatory response and trigger endothelial damage in preeclampsia [20].

In line with the study conducted by Than [14], cytoplasmic protrusion, microvilli membrane blebs, and shedding of membrane particles appeared to be immunopositive in this study. Upon IHC examination using anti-PP13 antibodies, an irregular syncytiotrophoblast basement membrane was seen, with discontinuous brush border membranes in several parts, protrusion, and membrane bleeding in several places in the preeclampsia group, especially in early onset. In normotensive pregnancy, the syncytiotrophoblastic basement membrane is still intact and has a regular surface. To see more clearly the ultrastructural picture of the syncytiotrophoblastic basement membrane, the researchers conducted a TEM examination. In the preeclampsia group, the discontinuity in the brush border membrane was more pronounced. In several places, necrotic shedding was seen, and in other places, prospective knots and syncytial knots were seen, while in the control group, the outline of the intact syncytiotrophoblast basement membrane with a neatly arranged membrane brush border was also clearly visible. In line with this study, previous studies showed that placental lesions and increased STBM shedding were seen more in early-onset preeclampsia compared to late-onset preeclampsia as soon as the clinical signs of preeclampsia appear [24,25]. Furthermore, the shedding of membrane particles are also immunopositive for PP13, which may explain the relatively increased maternal serum PP13 concentration in early-onset preeclampsia.

Meanwhile, the picture of chromatin condensation that occurs in the two preeclampsia groups seems to be relatively the same. It can be seen that the chromatin is clustered together with hydropic cytoplasm and is surrounded by fibrin and necrotic material (Figure 4). If this image is removed from the brush border membrane, it will form a wave-like aponeurosis shedding image (Figure 5). This material will later enter the maternal circulation and stimulate the maternal endothelial response.

### 4.5. The Pathomechanism of Aponecrosis, PP13, and Systemic Endothelial Dysfunction

Syncytiotrophoblast cells are believed to exhibit an imbalance between apoptosis and necrosis in preeclampsia. Because necrosis is more pronounced compared to apoptosis in preeclampsia, the membrane’s brush border region will exhibit more protrusions of necrotic material and syncytial knots. TEM examination reveals several subsets that are amassed along the vertical surface of the villi, with apparent chromatin condensation generating ring-like waves. Large amounts of STBM and necrotic particles will be released into the maternal circulation due to brush border membrane disruption, projecting cytoplasm, and the development of microvillous blebs [9], becoming one of the main causes of a specific maternal inflammatory response in preeclampsia. The amount of STBM and necrotic material discharged into the maternal circulation largely determines the moderate degree of endothelial dysfunction; the more STBM and necrotic material that enter the maternal circulation, the higher the risk of endothelial dysfunction.

This aponeurosis process involves a particular protein that the placenta produces, called Placental Protein 13. The amount of PP13 released into the maternal circulation increases along with the extent of the placental aponeurosis. Both early-onset and late-onset preeclampsia had greater levels of serum PP13. The LGALS13 gene is predominantly expressed by the placenta, primarily by the syncytiotrophoblast, because that affects the amount of PP13 released into circulation. This is the first of several factors that can lead to low expression of PP13 in the syncytiotrophoblast basement membrane and increased maternal serum concentrations of PP13. In addition, because placental LGALS expression was shown to be low in early-onset preeclampsia in earlier investigations, not much PP13 could be produced [14]. The major cause of elevated PP13 levels, which raise maternal blood concentrations of PP13 in the third trimester of early-onset preeclampsia, is, thus, thought to be excessive shedding of syncytiotrophoblast.

### 4.6. Strength and Limitation of the Study

In order to better understand early-onset and late-onset preeclampsia pathomechanisms, this study is the first to assess and compare the PP13 serum concentration and PP13 placental expression in preeclampsia and also the TEM image of syncytial knots and aponeurotic shedding. The placental syncytiotrophoblast was assessed using immunohistochemistry on all research samples; however, due to resource limitations, only a few representative samples were examined using TEM. Additionally, this study could not carry out statistical testing because it was not obvious how many knots were present in early-onset or late-onset preeclampsia. This is due to the limited number of resources available. Further study with a larger sample size is still needed to determine the role of maternal serum PP13 as a marker of systemic endothelial dysfunction.

## 5. Conclusions

PP13 expression in the syncytiotrophoblast was considerably lower in the third trimester of early-onset preeclampsia compared to late-onset preeclampsia and the control group. However, maternal serum concentrations of third-trimester PP13 did not differ among the early-onset preeclampsia, late-onset preeclampsia, and normotensive pregnancy groups. Placental syncytiotrophoblast aponeurosis was observed in the preeclamptic group but not in normotensive pregnancies.

## Figures and Tables

**Table 1:** Characteristics of subjects.

Characteristics	Early-Onset PE	Late-Onset PE	Control	*p* Value *
Age (years)	31.56 ± 7.47	29.37 ± 6.18	30.23 ± 2.32	0.381
Height (cm)	154.41 ± 4.76	153.96 ± 4.93	154.58 ± 5.93	0.907
Body Weight (kg)	72.89 ± 10.05	79.96 ± 10.14	69.04 ± 5.11	0.286
BSA (m^3^)	1.76 ± 0.14	1.74 ± 0.14	1.72 ± 0.08	0.423
BMI (kg/m^2^)	30.50 ± 3.37	29.91 ± 3.93	28.97 ± 2.77	0.263
Parity	1.33 ± 1.30	1.11 ± 1.34	1.15 ± 0.54	0.746
Gestational age (weeks)	31.37 ± 1.96	37.81 ± 1.90	38.00 ± 0.00	<0.001

Data reported as mean ± SD, α = 0.05; BMI: body mass index, BSA: body surface area, PE: preeclampsia; * one-way ANOVA.

**Table 2:** Maternal serum level of PP13.

Marker	Early-Onset PE	Late-Onset PE	Control	*p* Value *
PP13	260.8 (38.4–1233.4) ^+^	244.2 (80.4–722.6) ^++^	232.5 (34.6–476)	0.575

Data reported as median (min–max), α = 0.05; PP13: Placental Protein 13, PE: preeclampsia; ^+^ Mann–Whitney: no significant difference with late-onset PE, *p* > 0.05; ^++^ Mann–Whitney: no significant difference with control, *p* > 0.05; * Kruskal–Wallis.

**Table 3:** Expression of PP13 in placental syncytiotrophoblast.

Marker	Early-Onset PE	Late-Onset PE	Control	*p* Value *
PP13	3.38 ± 0.46 ^+^	4.06 ± 0.82 ^++^	4.51 ± 1.16	<0.001

Data reported as mean ± SD, α = 0.05; PP13: Placental Protein 13, PE: preeclampsia; ^+^ independent *T*-test: Significant difference with late-onset PE, *p* < 0.05; ^++^ independent *T*-test: significant difference with control, *p* < 0.05; * one-way ANOVA.

## Data Availability

The data presented in this study are available on request from the corresponding author.
